# The League of Mercy

**Published:** 1903-12-26

**Authors:** 


### HOSPITAL ADMINISTRATION.

### CONSTRUCTION AND ECONOMICS.

### THE LEAGUE OF MERCY.

The fourth annual meeting of presidents of the League of Mercy was held on the 18th instant in the Board Room of Charing Cross Hospital, when the chair was taken by Colonel Arthur Allen Owen (president of the Fulham district). Princess Alice of Albany (lady-president of the Epsom-Esher district) was present, attended by Miss Heron Maxwell. Amongst those present were: The Duchess of Somerset; Georgina, Countess of Dudley; Countess Cadogan; Dora, Countess of Chesterfield; Dowager Lady Lamington; Countess of Verulam; the Hon. Mrs. Freeman Thomas; Lady MacLean; Lady Faudel-Phillips; Lady Somerset; Marquess Camden; Earl of Kilmorey, K.P.; Lord Wolverton (hon. sec.); Sir Fitzroy MacLean, Bart., C.B.; General Sir William Stirling Hamilton, Bart.; Sir Henry Burdett, K.C.B. (hon. treasurer); Sir William Collins (hon. secretary); Sir M. M. Bhownaggree, K.C.I.E, M.P.; Colonel Sir Edwin Hughes; Lieut.-Colonel Sir Adelbert Cecil Talbot, K.C.I.E.; Colonel Knollys (registrar); Colonel Lockwood, M.P.; Mr. J. Harrison, M.V.O. (hon. secretrary); Mr. W. Bull, M.P.; Lieut. A. H. Tarleton, R.N.; Rev. H. Russell-Wakefield; Mr. J. A. Fyler, M.P.; Mr. R. C. Antrobus; Mr. A. Chancellor, J.P.; and others.

Letters expressing regret at inability to attend were received from:—H.R.H. The Duchess of Albany, H.H. The Princess Victoria of Schleswig-Holstein, The Countess Grosvenor, Lady Farquhar, The Duke of Argyll, K.T., G.C.V.O., The Earl of Clarendon, The Earl Cadogan, The Earl of Mansfield, Lord Farquhar, and others.

The following letter from Sir Arthur Bigge, received by the Hon. Secretaries, was read by the Chairman:

"I am directed by the Prince of Wales to express the gratification with which the Princess of Wales and himself, as Lady Grand President and Grand President of the League of Mercy, have learnt of the very successful results of the past year's work of the League of Mercy.

"Their Royal Highnesses take the opportunity of the annual meeting to ask that the expression of their best thanks may be conveyed to the Presidents and Lady Presidents of the various districts for their efforts, by which so large an increase in the subscriptions has been secured.

"It afforded their Royal Highnesses much pleasure to have an opportunity of making acquaintance with so many members of various grades in the League at Marlborough House last summer. Yours very truly,

(Signed) "Arthur Bigge."

On the motion of General Sir William Stirling-Hamilton, Bart., seconded by Lieutenant-Colonel Sir Adelbert Cecil Talbot, K.C.I.E., the Chairman, Deputy-Chairman and Hon. Secretary, and on the motion of Colonel Lockwood, M.P., seconded by Mr. Frederick Green, seven representative Members of the Executive Council were appointed.

Sir Henry Burdett (Hon. Treasurer) said that it was with very great pleasure he made his financial statement, because it not only showed considerable advance in the resources of the League, but also afforded gratifying evidence of the greater number of workers in and subscribers to the League. The advance made in many of the districts was very remarkable, and he would quote those which had made the greatest progress in the past year: Fulham had raised £1,017, as against £823 in 1902, an increase of £194; South Kensington came next with £794, as against £299, an increase of £495; St. George's, Hanover Square, £756, as against £696, an increase of £60; Guildford £640 (£500 of which was a donation from Lady Pirbright in memory of the late Lord Pirbright), against £290, having an increase of £350; North Paddington (a new district), £550; Westminster £490, as against £263; the Kingston and Richmond districts contributed £383 for Richmond and £217 for Kingston, being a total of £600 against £430. Tonbridge £414 as against £366, North St Pancras £407 as compared with £319, South Paddington £401 as against £168, Ealing (another new district) £379, Epsom-Esher raised £363 as against £33, Hampstead £343 as compared with £106, Whitechapel £325 against £214, West Marylebone £309 as compared with £212, Woolwich £243 as compared with £214, and Marylebone East had increased from £181 to £220. He thought that showed a very satisfactory state of affairs, and that satisfaction would be increased when the growth and development of the past year was taken into consideration. South Kensington stood first in the progress of the year, Epsom-Esher came second, Hampstead and South Paddington had almost tied, the one with an increase of £237, the other with an increase of £233, and Westminster ran them very close, showing an increase of £229. There was another very gratifying feature to note, viz., the districts had been largely added to. The figures he mentioned must be taken to a great extent as an estimate as the full accounts had not yet been received. The total amount received from all the districts in 1902 was £8,504. The estimated total for the year 1903 was over £12,700. That amounted to something like an increase of 50 per cent., and he only hoped that the League would follow its same prosperous course during 1904. The Executive Council had authorised him to pay £10,000 to King Edward's Hospital Fund as the contribution for the year 1903, which was an increase of £2,000 on the previous year and ten times as much as that contributed in the first year. This payment was sanctioned by the Grand President. Without the personal service which had been so freely given it would have been impossible to have had such an excellent result during what had been, in the business world, the most disastrous year England had seen for a long time.

Mrs. R. L. Harrison (President of the Fulham District) then gave a short statement of the work of the League in her district, speaking of the progress made amongst the poorer classes, and various suggestions were offered by several presidents with a view to the more extended and efficient working of the League.

Sir William Collins, M.D., one of the hon. secretaries, referred to the new districts which had been opened up during the past year. H.R.H. Princess Alice of Albany had become Lady President of the Epsom-Esher district with excellent results. H.H. Princess Victoria of Schleswig-Holstein had become Lady President of East Berks; and in North Kensington the Duchess of Somerset had accepted the position of lady president; whilst presidents and lady presidents had been appointed in the following amongst other districts: in Lewes, Lord and Lady Gage; in the Isle of Thanet, Lord and Lady Decies; in Chichester, Lady Edmund Talbot; at Horsham, General Sir William Stirling Hamilton and Lady Stirling Hamilton; at Epsom-Esher, Lieut.-Col. Adelbert Cecil Talbot; at Chelmsford, Lady Petre and Mr. Murray Ind; at Reigate, the Hon. Mrs. Leveson-Gower and Mr. Granville C. Leveson-Gower; at Rye, Captain Sir Augustus F. and Lady Webster. They much regretted the resignation of Mrs. Seymour Corkran as lady president of the Westminster district. With reference to entertainments, a garden party, at which over 1,000 workers of the League were present, was held last May at Marlborough House; a most successful concert had been promoted last June at Grosvenor House, from which a profit of £462 was realised; and only last Tuesday a dance, promoted by the Western Metropolitan districts, had taken place at the Empress Rooms. The Prince and Princess of Wales, as Grand President and Lady Grand President, had graciously promised to attend an entertainment which was proposed to be held in February next. Grants had been made during the past year to various local hospitals, after due inspection by members of the League. The speaker referred to a leading article which had appeared the other day in the columns of the *Times*, in which the authorities of King Edward's Hospital Fund and the Sunday Hospital Fund were recommended to devise some means by which they should appeal to the small tradesman and the prosperous artisan classes. He considered the League of Mercy supplied the machinery which was required for that purpose. Through the League no fewer than 60,000 men, women, and children, largely belonging to the humbler citizens, had been induced to take up work in support of the hospitals. In some cases subscriptions of such small sums as 2d. and 3d. were given. The remarkable growth of the League was shown by the fact that, whereas in 1899 (the first year of its existence) but one-fiftieth of the total revenue of King Edward's Hospital Fund was contributed by the League, this year the amount contributed was one-eighth of that revenue. The League, moreover, was the only source from which a constant, regular, and progressive increase of support came to the King's Fund.

Many of their lady and gentlemen workers had had great difficulties with which to contend. That day was, as it were, their harvest festival, and he was certain that many who had gone forth weeping with their precious seed had returned with joy, bringing their sheaves with them.

A vote of thanks, moved by Sir Fitzroy D. Maclean, and seconded by Mr. H. L. Jephson, was passed to the authorities of Charing Cross Hospital for their kindness in granting the use of the board-room for the meeting; and a vote of thanks to the Chairman, moved by Colonel Sir Edwin Hughes, and seconded by Mr. W. J. Bull, M P., brought the meeting to a close.

